# Biomedical and Tissue Engineering Strategies to Control Foreign Body Reaction to Invasive Neural Electrodes

**DOI:** 10.3389/fbioe.2021.659033

**Published:** 2021-05-25

**Authors:** Manuele Gori, Gianluca Vadalà, Sara Maria Giannitelli, Vincenzo Denaro, Giovanni Di Pino

**Affiliations:** ^1^Laboratory for Regenerative Orthopaedics, Department of Orthopaedic Surgery and Traumatology, Università Campus Bio-Medico di Roma, Rome, Italy; ^2^Institute of Biochemistry and Cell Biology (IBBC) - National Research Council (CNR), Rome, Italy; ^3^Laboratory of Tissue Engineering, Department of Engineering, Università Campus Bio-Medico di Roma, Rome, Italy; ^4^NeXT: Neurophysiology and Neuroengineering of Human-Technology Interaction Research Unit, Università Campus Bio-Medico di Roma, Rome, Italy

**Keywords:** neural electrodes, foreign body reaction, coatings, biomaterials, hydrogel, tissue engineering, microfluidics, nanofabrication techniques

## Abstract

Neural-interfaced prostheses aim to restore sensorimotor limb functions in amputees. They rely on bidirectional neural interfaces, which represent the communication bridge between nervous system and neuroprosthetic device by controlling its movements and evoking sensory feedback. Compared to extraneural electrodes (i.e., epineural and perineural implants), intraneural electrodes, implanted within peripheral nerves, have higher selectivity and specificity of neural signal recording and nerve stimulation. However, being implanted in the nerve, their main limitation is represented by the significant inflammatory response that the body mounts around the probe, known as Foreign Body Reaction (FBR), which may hinder their rapid clinical translation. Furthermore, the mechanical mismatch between the consistency of the device and the surrounding neural tissue may contribute to exacerbate the inflammatory state. The FBR is a non-specific reaction of the host immune system to a foreign material. It is characterized by an early inflammatory phase eventually leading to the formation of a fibrotic capsule around intraneural interfaces, which increases the electrical impedance over time and reduces the chronic interface biocompatibility and functionality. Thus, the future in the reduction and control of the FBR relies on innovative biomedical strategies for the fabrication of next-generation neural interfaces, such as the development of more suitable designs of the device with smaller size, appropriate stiffness and novel conductive and biomimetic coatings for improving their long-term stability and performance. Here, we present and critically discuss the latest biomedical approaches from material chemistry and tissue engineering for controlling and mitigating the FBR in chronic neural implants.

## Introduction

Since scientists started to invasively study the function of the central nervous system (CNS) and peripheral nervous system (PNS), single electrodes, and later on electrode arrays, have been implanted to record neuronal activity and to stimulate single or groups of neurons to artificially induce their activation, in light of decoding their functions.

Once study protocols moved from acute tests to chronic implantations and the safety of implants performed in primates suggested the possibility to move to studies in humans, a further possible application of invasive neural electrodes, beside that to investigate neuronal functions, became concrete. Electrodes started to be employed to decode subject motor intention and, bypassing neural or osteo-muscular lesions, to artificially interface the nervous system to the external environment.

When this happened, neural interfaces -often named brain to computer or to machine interfaces- and the field of neuroprosthetics were born. Depending on the site and the subject receiving the implant, electrodes can also be interfaced with sensory area and fibers and, by relaying afferent streams of information, convey artificial sensory feedback.

Insofar, some applications for stimulating neural electrodes, particularly deep brain stimulation (DBS) and cochlear implants, have gained the maturity to be commonly applied in clinical practice. Other applications targeting a more spatially-selective information exchange, such as cortical or peripheral nerve implants, are very-promising, yet still in a developmental phase. Their not-complete maturity is mostly due to the lack of long-lasting stability of their performance over time, mainly because of the reaction that the body mounts around them. This factor hampers to a less extent cochlear electrodes, because they do not penetrate the neural structures, and DBS, because these electrodes do not need to achieve the level of stimulation selectivity needed by information exchange. The long-term functionality and longevity of cochlear implants and deep brain stimulators have already been widely demonstrated (Deuschl et al., 2006; Woeppel et al., 2017).

Contrarily, the use of invasive multichannel electrodes, implanted within stump peripheral nerves to control cybernetic hand prostheses, is an application field of neural interfaces where electrodes should achieve an intimate contact with neural fibers required to reach a reliable information transmission, and where implantable solutions seem to favor exchange selectivity.

Since peripheral nerves contain both motor and sensory fibers, peripheral nerve electrodes can achieve proper bidirectional communication through the use of a single device by stimulating afferent axons (Xavier and Jaume, 2014).

Regained sensory feedback from hand prosthesis has the potential to improve motor control (Valle et al., 2018; Zollo et al., 2019), discrimination abilities (Raspopovic et al., 2014), and to reverse aberrant brain plasticity triggered by the amputation (Rossini et al., 2010; Di Pino et al., 2012, 2014; Ferreri et al., 2014; Serino et al., 2017).

Unfortunately, the standard control systems of prosthetic limbs rely on surface electromyogram, which is mainly limited by problems of high latency, as well as low specificity and robustness in long-term implants (Anderson and Weir, 2019). Although some of the current peripheral nerve interfaces can shorten latency and provide single axon specificity, their performances tend to degrade with time due to the biological response of the organism to the electrode, which is triggered by the damage provoked by the implant procedure itself (Anderson and Weir, 2019). The body tends to insulate and exclude the foreign material from the surrounding microenvironment, leading to scar tissue growth around the device that is made of a fibrous capsule.

In the conductive surface, the dielectric constant, dissipation factor and dielectric loss factor rise with the increase of the capsule thickness. The increase of the electrical impedance is proportional to the development of the fibrotic tissue, which determines difficulties to distinguish the signal from background noise (Szostak et al., 2017) and, eventually, the drop of stimulation and registration capacities (Guadarrama-Santana and Garcia-Valenzuela, 2007; Jayamani et al., 2014).

The immune-mediated response responsible for the capsule growth is known as Foreign Body Reaction (FBR). FBR reduction over time is probably the main challenge for future neural electrode applications in neuroprosthetics to extend the reliability of the interface (Lotti et al., 2017).

The aim of this review is to analyze the latest tissue engineering strategies and biomedical approaches for controlling and evading FBR around implantable interfaces.

Although the FBR process can occur in any living tissue implanted with foreign material, such as molecularly engineered surfaces and medical devices (Anderson et al., 1999; Luttikhuizen et al., 2006), we will restrict our field of investigation and focus the review toward intraneural electrode applications to interface robotic prosthetic limbs.

We analyze factors supposed to be the main causes of acute and chronic neural tissue reactions, such as scarce biocompatibility, excessive size, poor flexibility, reduced electrical properties, low compliance, mechanical mismatch and micromotion.

Finally, we examine the shortcomings of current electrode-producing technologies and discuss possible cutting-edge solutions for the development of promising alternatives to the present intraneural interfaces. Strategies and technologies analyzed in light of the specific application we pursue could be potentially tailored to any electrode inserted in the CNS or PNS, and interfaced with different artificial devices.

## Molecular Mechanisms and Cellular Components of the FBR

**FIGURE 1:**
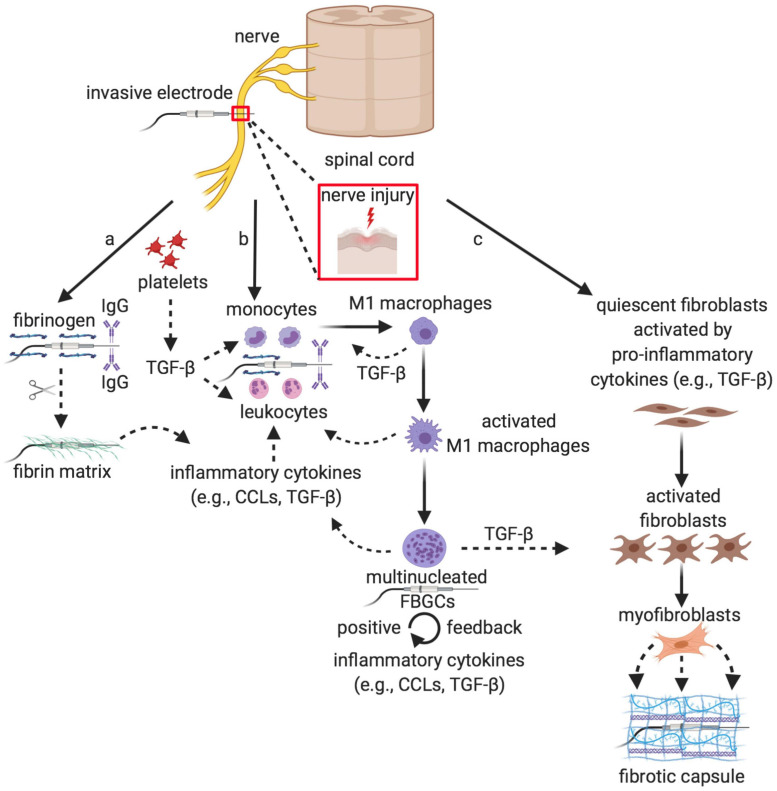
Onset, progression and resolution of the Foreign Body Reaction. Sequence of cellular events of the non-specific immune response elicited by the biomaterial surrounding the invasive electrode implanted into the nervous tissue, which is perceived as a nerve injury: (a) onset, similarly to the wound healing, the adsorption of blood and plasma proteins [in particular, fibrinogen and antibodies (IgG), which will be recognized by the white blood cells of the immune system, and the complement system providing specific binding sites and chemoattractants for circulating leukocytes and monocytes] to the surface of the implant leads to the second step of the process, (b) the progression of the FBR, during which leukocyte and monocyte extravasation that is due to the influence of various chemokines, such as TGF-β, promotes their attraction and adhesion to the electrode surface. Recruited monocytes differentiate into activated M1 macrophages that fuse together into multinucleated FBGCs, which carry out multiple functions including: the increase of the inflammatory response both through a positive feedback mechanism (mainly *via* additional TGF-β production) and through the recruitment of further monocytes and macrophages, the digestion of the electrode surface while promoting the recruitment of the fibroblasts and their activation to myofibroblasts in the last step of the process, (c) the resolution of the FBR, during which the myofibroblasts secrete the different ECM components around the implant that are responsible for the formation of the fibrotic capsule, which ultimately isolates the electrode from the surrounding tissue. IgG, immunoglobulin G; CCLs, CC chemokines; TGF-β, transforming growth factor β; FBGCs, foreign body giant cells. Created with BioRender.com.

In a living tissue or a nerve, any implantation of foreign material, including advanced biomaterials that surround an invasive electrode, triggers an unbalanced biological reaction (i.e., characterized by scarce wound healing and chronic inflammatory state) of the non-specific immune system, known as FBR, which is the natural protection mechanism of the host to the foreign body (Anderson et al., 1999; Luttikhuizen et al., 2006). This complex host reaction (Figure 1) consists in a sequential and orderly cascade of molecular events that involves adhesive blood and plasma proteins, tissue and infiltrated inflammatory cells, and inflammatory cytokines.

The first step (onset), which is similar to wound healing, upon foreign body implantation is the adsorption of blood and plasma proteins, such as fibrinogen, fibronectin, albumin and antibodies to the implant surface (Andrade and Hlady, 1987; Jenney and Anderson, 2000). The type of the proteins adsorbed and the progression of the FBR depend on the surface shape, chemistry composition and charge (Tang and Eaton, 1993; Hunt et al., 1996; Thull, 2002). In the second step (progression), the adsorbed protein layer and its composition in turn promote monocyte and leukocyte extravasation, attraction and adhesion to the surface, along with the activation of the coagulation cascade (Richardson et al., 1976; Smiley et al., 2001; Szaba and Smiley, 2002).

Fibrinogen is hydrolyzed to fibrin that creates a sort of matrix able to attract circulating leukocytes and local macrophages around the implanted surface under the chemoattractive influence of different chemokines (Tang et al., 1998; Smiley et al., 2001; Tao and Kobzik, 2002; Lishko et al., 2004). At the onset of the FBR and during its progression, circulating platelets first and macrophages then secrete transforming growth factor β (TGF-β). This pivotal cytokine serves as chemoattractant and activator of monocytes, besides being responsible for the continuum of the inflammation and its exacerbation until fibrosis (DiPietro et al., 1998; Crowe et al., 2000). Leukocytes express and secrete a series of other inflammatory cytokines, such as CCL2, CCL3, CCL5, which are the principal players involved in the recruitment of blood-borne monocytes and local macrophages in the foreign body microenvironment (DiPietro et al., 1998; Hancock et al., 2000; Ono et al., 2003; Armstrong et al., 2004). Afterward, extravasated monocytes differentiate to macrophages that, once activated under the stimulation of activated T cells, fuse together to form multinucleated foreign body giant cells (FBGCs). FBGCs start releasing further inflammatory cytokines, thus boosting the inflammatory response through a mechanism of positive feedback, giving rise to a chronic inflammation (Anderson, 2000; Kyriakides et al., 2004). This cell recruitment from the bloodstream is allowed by vasodilatation and increase of vessels permeability, which is induced by the platelet release of the angiogenic cytokine vascular endothelial growth factor (VEGF) (Banks et al., 1998; Ferrara et al., 2003). The biological activity of the FBGCs represents a hallmark of the FBR, as it is aimed to protect implanted tissue against the foreign body, mediating its surface damage and digestion through the release of various proteases and acids (Kyriakides et al., 2004). In the last step of the process (resolution), macrophages play a key role *via* the production of TGF-β. This multifunctional cytokine has a paramount importance as it will stimulate the fibroblast-mediated extracellular matrix (ECM) production, while reducing at the same time inflammation (Bellingan, 1996; Ashcroft, 1999). Thus, the role of the recruited macrophages is to promote further monocyte and macrophage recruitment and to stimulate the growth and differentiation of quiescent fibroblasts to myofibroblasts. Myofibroblasts are eventually responsible for the massive production and secretion of ECM components, including collagen I, collagen III, fibronectin and proteoglycans that give rise to the dense fibrotic capsule around the implanted electrode (Luttikhuizen et al., 2006; Anderson et al., 2008; Ward, 2008). In the very final stage of the process, the capsule becomes impermeable to the non-specific immune system and to many chemicals, including some therapeutic inhibitors of inflammation, and responsible for the augmentation of the electric impedance and progressive isolation of the implanted device, impairing its long-term functionality (Anderson et al., 1999, 2008; Luttikhuizen et al., 2006).

## Intraneural vs. Extraneural Electrodes in FBR

To interface with a peripheral nerve invasive intraneural and extraneural electrodes can be employed. Among intraneural electrodes, the most used are Multielectrode arrays (MEAs), Longitudinal Intra-Fascicular Electrodes (LIFEs) and Transverse Intrafascicular Multichannel Electrode (TIME) (Yoshida and Stein, 1999; Branner et al., 2004; Badia et al., 2011; Yildiz et al., 2020). The extraneural electrodes developed to interface with peripheral nerve are cuff electrodes (Navarro et al., 2001; Ortiz-Catalan et al., 2013) and Flat Interface Nerve Electrodes (FINEs) (Tyler and Durand, 2002; Freeberg et al., 2020).

Intraneural electrodes should offer a better signal-to-noise ratio during neural recording and the reduced current intensity necessary to reach the appropriate nerve stimulation (Navarro et al., 2005). Nonetheless, being implanted within the nerve, these interfaces are traumatic for the surrounding tissue triggering an early inflammatory response caused by the injury of the vascularized connective tissue. Indeed, as the electrode proximity to the nerve increases, a higher selectivity of neural recording of the signal and stimulation can be obtained. However, the formation of the fibrotic capsule around the interface reduces recording and stimulation long-term stability compared to extraneural electrodes (Rossini et al., 2010; Badia et al., 2011, 2016; Lotti et al., 2017).

The chronically implanted devices stimulate the aforementioned multistep cascade of foreign body response, ending in scar tissue formation and electrode encapsulation, and thus in the need of increased currents (i.e., power consumption) to maintain appropriate nerve stimulation due to a progressive increase of the electrical impedance. The most frequently used metals for the fabrication of neural electrodes are gold, tungsten, platinum (Pt) and Platinum-Iridium (Pt-Ir) alloy, with Pt being considered the preferred choice for long-term neuroprosthetic applications due to its electrochemical stability, safety, resistance to corrosion and limited reactivity within a tissue environment (Brummer et al., 1983; Geddes and Roeder, 2003; Merrill et al., 2005; Polikov et al., 2005). However, the stiffness of Pt has a traumatic impact on the surrounding soft neural tissue (Green et al., 2012), causing a shear stress that over time induces an inflammatory reaction, which can be further stimulated by the tissue movements and electrode micromotion (Rousche et al., 2001; Leach et al., 2010). In addition, another weakness of Pt and other metallic electrodes is due to their fabrication, which is usually performed with smooth surfaces that do not allow complete nervous tissue adhesion and integration. As a result, immune cells may invade the remaining space between device surface and target nerve in the implanted area, fostering the FBR (Aregueta-Robles et al., 2014). Therefore, the strength of the implant-tissue integration is influenced by the presence of FBGCs and monocytes/macrophages (Fink et al., 2008). On the other hand, manufacturing excessively rough surfaces may risk increasing the local strain and producing friction forces, thereby causing tissue damage. It is also known that rougher surfaces are able to alter cell adhesion, growth, activation and behavior (Fink et al., 2008; Gamboa et al., 2013; Hulander et al., 2013) including macrophage fusion (Chen et al., 2010), although these effects depend on the different cell types as well as on the materials used and their fabrication methods. Consequently, the right compromise should be sought between the optimal flatness, smoothness and suitable roughness that meet the texture of the nerve tissue, thus avoiding local insults and hazardous damages that could trigger inflammation and a deranged wound healing process. Because of these intrinsic limitations in metallic electrode efficiency, the continuous search for valid alternatives and chemical modifications to material composition is encouraged. For example, electrodes can be coated with conductive and soft polymers, like a core of flexible and insulating polyimide with metallic tracks of Pt or Pt-Ir, as detailed below. Such a strategy can be adopted for mitigating the stiffness disparity between device and host tissue and for relieving the biological rejection of the nerve tissue (Geddes and Roeder, 2003; Merrill et al., 2005; Polikov et al., 2005).

**FIGURE 2:**
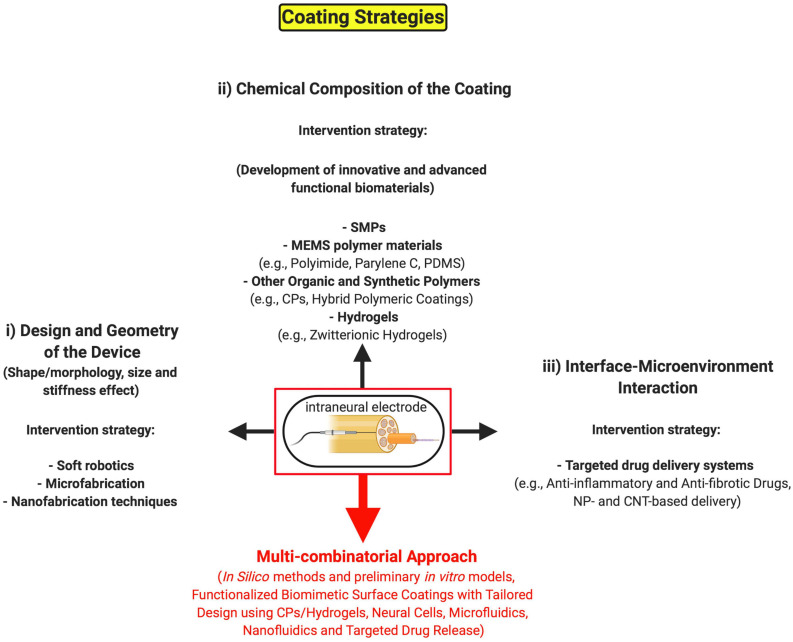
Possible coating strategies of invasive neural implants to minimize the long-term FBR. Schematic representation of the main tissue engineering strategies for coating intraneural electrodes against the FBR: (i) the long-term stability and performance of invasive interfaces can be enhanced through the manipulation of the electrode shape, size, geometry, flexibility and surface roughness to create minimally invasive neural implants by leveraging on micro- and nano-manufacturing methods. (ii) An alternative intervention strategy consists in the development of novel chemical coatings, making use of advanced functional biomaterials, as biocompatible surfaces. (iii) Lastly, the functionalization of the coating with therapeutic drugs and, accordingly, innovative drug-delivery systems may help better integrate and tolerate the invasive device by the host nervous tissue. All of the above intervention strategies could be hopefully integrated into a unique multi-combinatorial approach (red arrow) in the next future. SMPs, shape memory polymers; MEMS, micro-electro-mechanical systems; PDMS, poly(dimethylsiloxane); CPs, conductive polymers; NP, nanoparticle; CNT, carbon nanotube. Created with BioRender.com.

So far, diverse strategies are being pursued (Figure 2) to create minimally invasive neural implants that may address the FBR issue and guarantee their long-term use, which can be summarized as follows: (i) working on the design and geometry of the device (such as surface roughness, electrode shape, size, and flexibility); (ii) working on the chemical composition of the coating material to develop novel organic and synthetic polymer substrates that can be tolerated much better by the host tissue. Finally, another important approach consists in (iii) working on the interaction between interface and microenvironment for controlling the local delivery of therapeutic molecules (e.g., anti-inflammatory and anti-fibrotic drugs) making use of functionalized biomimetic and biodegradable coatings.

### Working on the Design and Geometry of the Electrode

The shape and topography of medical-grade polymers implanted in animal models profoundly influences the FBR at the implant surface, with the broadly accepted experimental outcome that circular and smooth surfaces, in intramuscular and percutaneous implants, minimally affect the aggressive behavior of macrophages (Matlaga et al., 1976; Salthouse, 1984). The use of flexible implants of multifunctional polymeric fibers (Canales et al., 2015), and microfabrication of the electrode shape with a new flexible sinusoidal design and a 3D spheroid tip that reduce local strain and tissue damage caused by micromotion (Sohal et al., 2014) may represent alternative strategies to gain some mechanical benefits, without remarkably modifying the size of neural implants, and improve their *in vivo* longevity and recording performances. The importance to focus on the coating stiffness and geometric configuration (i.e., size effect), to reduce the mechanical mismatch between chronic implanted electrodes and neural tissue, has been highlighted by a recent work of Spencer et al. (2017). They investigated the ability of soft polyethylene glycol dimethacrylate (PEG-DMA) hydrogel coatings, compared to hard implants of identical diameter, to reduce chronic glial scar formation on the surface of neural probes in rodent brains, by lowering the local strain and diameter (from 400 to 150 μm) of the coating. The authors suggest that a similar technique could be adapted to coat more complex geometries through a dip coating, or spray coating method, including electrodes made of various materials, such as metal, silicone and polymer implants, by slightly changing the chemistry.

The strategy of coating neural electrodes with hydrogels of PEG and PEG-based copolymers, leveraging their high versatility, low-fouling and bioinert properties, has long been used with moderate success in many studies (as reported with various examples in the subsection “other advanced biomedical materials”) (Wichterle and Lím, 1960; Rao et al., 2011; Gutowski et al., 2015; Heo et al., 2016), although some limitations that were somehow addressed combining PEG with other polymers. However, PEG shows high susceptibility to oxidative damage *in vivo*, it may activate severe immune response, and its functionalization is usually troublesome, thereby limiting its application for neural interfaces that require long-term stability (Ostuni et al., 2001; Ward et al., 2002; Knop et al., 2010). Likewise, poly(2-hydroxyethyl methacrylate) (PHEMA), which is, together with PEG, the most widely used coating material for implantable devices (Campioni et al., 1998; Ratner, 2002) is susceptible to non-specific protein adsorption, and eventually to fibrotic encapsulation (Zhang et al., 2017), thereby raising the same problems faced with PEG for the extended stability over time and for restraining an immune reaction. Instead, hydrogels made of zwitterionic polymers, such as poly(carboxybetaines), poly(sulfobetaines), and poly(phosphobetaines) (Chen et al., 2005; Jiang and Cao, 2010; Sin et al., 2014a, b) are biocompatible and highly hydrated materials, showing anti-inflammatory and ultralow-fouling characteristics *in vivo*, which hold great potential to reduce FBR a way better than PEG hydrogels (Jiang and Cao, 2010; Zhang et al., 2013; Wu et al., 2018), as further discussed below in the hydrogel section.

In the last decades, many endeavors have been made in different biomedical and clinical frameworks, merging microengineering and material chemistry skills with molecular biology knowledge, to modify the physicochemical features of implanted interfaces and tuning their structural and surface features with the aim to control the FBR and increase their neurocompatibility. For example, it was initially proposed the use of the focused ion beam technology as high precision machining technique to create and modify the surface morphology of the interface material, up to nanometric scale, by controlling the ion milling of the substrate or its coating in three dimensions, and thereby modulating *in vitro* the neural cell adhesion (Raffa et al., 2007). Afterward, other promising solutions developed for patterning the design and morphology of the surface are briefly summarized as follows: the generation of combinatorial libraries of cationic polymer coatings in mice (Ma et al., 2011); the intramuscular implantation, in rat spinotrapezius muscles, of biodegradable poly(l-lactide-co-d/l-lactide) (PLA), as membranes and uncoated electro-spun fiber meshes with a positively charged plasma-polymer coating, to alter material morphology (Lucke et al., 2018). Finally, the development of a method to control surface porosity of poly(2-hydroxyethyl methacrylate-co-methacrylic acid) (pHEMA-co-MAA) hydrogels, consisting in the fabrication of parallel channels interconnected to a micrometer-sized spherical pore network (Madden et al., 2010). These surface-modified scaffolds were able to increase neovascularization and reduce the inflammation and tissue scarring. This last work represents another smart approach to control channel size and spacing of a functionalizable surface, which can be achieved by varying the dimensions of the microsphere templates. With regard to changing the geometry of the electrode material, the anionic polysaccharide, alginate, is a naturally-derived polymer able to form biocompatible hydrogels, with the addition of divalent cations, to encapsulate cells and materials for biomedical applications (Lee and Mooney, 2012; Veiseh et al., 2015; Vegas et al., 2016; Bochenek et al., 2018). Semi-permeable alginate spheres have been developed since long time as a common tissue engineering strategy to isolate implanted biological material from the effect of the local immune cells, thus reducing the FBR *in vivo* (Chang, 1964; Lim and Sun, 1980; Veiseh et al., 2015). Significantly, in one of these works, the authors showed for the first time the importance of the size and spherical geometry not only for the SLG20 alginate hydrogel encapsulation of pancreatic islets, but also for stainless steel, glass and polystyrene spheres on the fibrotic response in immunocompetent and fibrosis-prone rodent and non-human primate models (Veiseh et al., 2015). They tested different sizes and time windows, including a chronic time-point (i.e., 6 months), for transplanted grafts encapsulated with the SLG20 alginate capsules and found the 1.5 mm-sized spheres as the ideal geometry to protect grafted cells and surfaces from macrophage activation and fibrosis compared to smaller spheres. In conclusion, they demonstrated that size (1.5 mm in diameter or greater) and spherical shape, rather than stiffness, of alginate hydrogels as well as ceramic, metal and plastic surfaces represent critical features for obtaining prolonged biocompatibility and for resisting to fibrosis rejection. So, this biomaterial design strategy is potentially applicable to intraneural interfaces although, at present, such dimensions are not always achievable for all the intraneural electrodes. Instead, the strategy proposed by Rubehn and Stieglitz (2010) consists in a novel 3D design of a spiked ultraflexible neural (SUN) interface that integrates spiked structures for intrafascicular nerve recording from the PNS with an ultraflexible substrate, thereby enabling a unique conformal interface to the target nerve. The advantage is represented by the features of the material used, which is an insulating polyimide substrate that does not cause excessive inflammation. Hitherto, this new sensor model has been used only in acute animal experiments, whereas for chronic implantations important challenges still remain to be faced such as, among others, the FBR with fibrotic scar tissue that could displace the electrode from its original position and thus jeopardize the quality of the neural signal (Rubehn and Stieglitz, 2010; Wang J. et al., 2018).

Overall, it seems very important to modify the electrode surface with more smooth and circular shape, without major changes in size, to reduce both the local strain of the material and the mechanical mismatch between the device and the host tissue. The consequences of altering the surface topography, in particular the effect of the roughness, are still debated and quite complex to understand (Fink et al., 2008). In fact, topography-induced changes seem to affect macrophage behavior (e.g., cell adhesion, fusion and cytokine secretion *in vitro*) in the FBR to diverse polymer surfaces (Chen et al., 2010). Furthermore, the continuous search for novel 3D surface design of the device, with coatings at high flexibility, which can be able to adapt to the microenvironment, shaping themselves to the nervous tissue would represent a plus for improving more and more the implant integration. To this aim, 3D bioprinting of hydrogels and thin-film deposition technologies of biocompatible and soft polymers will facilitate the task.

## Modifications of the Intraneural Electrodes by Integrating Soft Robotics, Microfabrication of Microfluidic Systems and Carbon Nanotubes

In the research field of neural electrodes and probes continuous efforts are being made in search of smaller and more flexible devices to reduce the trauma caused by their insertion and, in turn, the biological tissue response (chronic inflammation and fibrosis), leveraging on micro- and nano-fabrication techniques. Recently, an innovative soft robotics approach has been devised to mitigate the FBR by controlling fluid flow and shear stress perceived by the host cells (Dolan, 2019). In a rat model, the authors implanted subcutaneously a milliscale dynamic soft reservoir (DSR), surrounded by an actuatable polyurethane membrane, and modulated the biomechanics of the biotic-abiotic interface *via* tunable pressure. After 14 days, an important reduction in the number of αSMA+ myofibroblasts and in fibrotic encapsulation of the implantable device was observed through histological and immunohistochemical analysis. Furthermore, as an example of a proof-of-concept study using a porous and permeable actuating membrane, they were also able to regulate therapeutic delivery of epinephrine, used as a model pharmacological agent, to test its functional effect in the adjacent tissue. Hence, the presented DSR may have the potential to be integrated into intraneural electrodes for an extended period to modulate the inflammatory and fibrotic response, making it a promising tool also for future neural applications. In fact, the design of the platform can be easily modified and tailored to be integrated into diverse types of implantable devices through its incorporation into a thin matrix that can be part of an intraneural electrode. In the past decades, flexible polymer-based microelectrodes have been developed also for neural prosthetic devices (e.g., testing different device size, shape, surface smoothness and structural stiffness) taking advantage of microfluidic and micromachining techniques (Szarowski et al., 2003; Lee et al., 2004; Polikov et al., 2005). Despite these microelectrodes provide multiple and high-quality stimulation and recording sites, the lack of long-term stability has been reported due to the neural tissue reaction and scar formation following extended microelectrodes implantation (Lee et al., 2004). To overcome this limitation, scientists sought to integrate microfluidic channels into flexible microelectrodes combining different techniques for achieving controlled delivery of anti-inflammatory and anti-fibrotic drugs through the microchannels, as further reviewed in Section “Interface-Microenvironment Interaction.” However, micromachining of the electrode polymer through a lamination technique (Metz et al., 2004), micromolding and thermal bonding of the polymer (Ziegler et al., 2006), combined electrochemical deposition of conductive polymer and drugs on the electrode (Wadhwa et al., 2006), turned out to be complex and expensive for a large-scale use. Hence, novel microelectrodes, combining thin-film fabrication with poly(dimethylsiloxane) (PDMS) molding and a more rapid, easy, and cost-effective bonding technique, enabled long-term drug release for a more stable recording performance (Gao et al., 2013). A new hybrid cuff electrode that integrates microelectrodes, for recording and stimulation, embedded within microfluidic channels for drug delivery is an example of flexible thin-film polymer device fabricated *via* surface micromachining techniques on a temporary silicon wafer carrier (Elyahoodayan et al., 2020). The electrode was designed and developed to improve fascicular selectivity and sensitivity in rat sciatic nerves following minimal handling during surgical implantation. Its main advantage is represented by the combined possibility to acutely stimulate, record and deliver lysing drugs, to remove connective tissue (i.e., epineurium layer) that separates electrodes from nerve fibers, and neurotrophic factors that promote axonal sprouting from the exposed fibers. Nevertheless, the authors stated that future studies will be necessary for functional testing in prolonged implant conditions to check for chronic electrophysiological recording as well as nerve health and interface stability after collagenase delivery to verify possible levels of axonal inflammation and fibrosis. Regarding novel and advanced production methods of microelectrodes, a great deal of interest has recently emerged in the additive manufacturing techniques, a versatile and powerful tool to overcome various shortcomings of conventional lithography techniques. Additive manufacturing of microelectrode arrays or microneedle arrays provides a novel, quick and low-cost method to fabricate custom-shaped electrochemical devices, by rapid prototyping, for a wide range of applications (Yang et al., 2016; Morrison et al., 2019; Soltanzadeh et al., 2020). For example, the manufacturing method performed by an aerosol jet technology, for the fabrication of the microelectrode arrays used in a biosensor platform for electrochemical measurements, was based on the use of a silver nanoparticle (NP) ink and a UV-curable polymer (Yang et al., 2016). Instead, in another work, compared to microfabricated microneedle arrays, 3D-printed arrays, made of an amorphous polymer of acrylonitrile-butadiene-styrene, showed almost identical geometric properties and equivalent performance with high frequency biosignals (such as in electromyogram recordings), whereas for recording low frequency signals they turned out to be not suitable (Soltanzadeh et al., 2020). However, in these works, only preliminary and short-term tests were run to measure their functionality (e.g., electrical stimulation in mouse brain, signal recording ability and impedance characteristics) either in human subjects in a non-invasive manner (Soltanzadeh et al., 2020), or in mice (Morrison et al., 2019) and as electrochemical laboratory biosensors (Yang et al., 2016), thus requiring further and deeper *in vivo* investigation to establish the real advantages and drawbacks of 3D-printed microelectrodes and the biocompatibility of the materials used before their clinical application.

To date, microelectrode technologies present important limitations mainly due to the stiffness mismatch between metals or micromachined silicon, used for electrode microfabrication, and surrounding tissue, particularly soft brain tissue (Winslow and Tresco, 2010). Thus, the mismatch results in fibrotic encapsulation of the microelectrode in chronic implants (Polikov et al., 2005). Furthermore, the problem of controlling possible micromotion of the interface that can change its position in the tissue may also gradually increase the inflammatory reaction (Gilletti and Muthuswamy, 2006). Similar issues can occur with chronic implants of microfabricated peripheral nerve devices. Thus, another group developed a novel fluidic microdrive technology to implant and microactuate ultraflexible electrodes, with a parylene-coated core of carbon nanotube (CNT) fibers, in animal models that could find useful applications also in peripheral nerves (Vitale et al., 2018). Indeed, following fluidic implantation into the nervous tissue, the authors were able to perform electrophysiological recordings, enhancing the stability of the device without the need of increasing the stiffness and thickness of the microdevices, and thus preventing also the onset of inflammatory responses. Fluidic microdrives were fabricated in PDMS by conventional replica molding technique and the microelectrodes insertion was obtained *via* viscous drag force due to the finely controlled liquid flow in the microfluidic channel, limiting tissue damage at a negligible extent. Such brilliant strategy could be further implemented for peripheral nerve electrodes, envisioning exciting opportunities for their chronic implants. Wireless and flexible film-based ion-selective electrodes (ISEs) have also been recently developed as miniaturized systems for performing highly sensitive and non-invasive measurements (Lim et al., 2020). These sensor systems, made of carbon–polymer composite transducers integrated onto a flexible circuit, enable ions detection in body fluids with high accuracy and selectivity and for prolonged lifetime, showing great potential for their application also in health studies and clinical systems. Another recent approach to drastically reduce the risk of alteration of the performance of the transducer material used for sensors and electrodes, was the development and characterization of solid contact ion-selective electrodes using novel composite material (Kałuża et al., 2019). The formulation of the present nanocomposite was based on multi-walled carbon nanotubes (MWCNTs) and poly(3-octylthiophene-2,5-diyl) (POT), with the immobilization of the polymer on the carbon nanostructures, preventing its spontaneous and unwanted partition to the membrane phase. The obtained sensors were characterized with good performance, high conductivity as well as high stability of potential readings over time. Nevertheless, although the remarkable electrical and physical properties of CNTs that can be exploited for enhancing the functionality of metallic electrodes (Aregueta-Robles et al., 2014), the main concern for their long-term use *in vivo* remains related to their cytotoxicity and to the risk of causing intracellular damages. Indeed, because of their elevated stiffness and reduced size (Krishnan et al., 1998), CNTs can easily penetrate cellular membranes (Kagan et al., 2006; Gilmour et al., 2013) and damage nuclei and cytoplasmic organelles. Additionally, they are known to be cytotoxic at high concentrations in different cell types (Bottini et al., 2006; Tian et al., 2006). In spite of such significant risks, which need to be carefully evaluated before clinical applications, nanoscale features of CNTs enable their escape from the immune system surveillance, thereby providing an undoubtedly appealing resource for the future development of innovative intraneural electrodes. A summary of the intervention strategies based on the design and geometry of the electrode with representative examples is reported in Table 1.

**TABLE 1:** Intervention strategies based on the design and geometry of the electrode.

(i) Design and geometry		

Features	Examples	References
Size effect	PEG-DMA hydrogel coatings and deep and spray coating method	Spencer et al., 2017
	PEG-based coatings	Reviewed in Knop et al. (2010) (Wichterle and Lím, 1960; Rao et al., 2011; Gutowski et al., 2015; Heo et al., 2016; Lee et al., 2017)
	PHEMA-based coatings	Reviewed in Ratner (2002) (Campioni et al., 1998; Jhaveri et al., 2009; Zhang et al., 2017)
Surface morphology	FIB technology as machining technique to modify surface morphology	Raffa et al., 2007
Shape	Flexible implants of multifunctional polymeric fibers	Canales et al., 2015
Design and topography	Physical properties, surface micro-/nano-topography and surface chemistry modifications	Reviewed in Ware et al. (2013) (Anderson et al., 1999; Thull, 2002; Fink et al., 2008; Chen et al., 2010; Gamboa et al., 2013; Hulander et al., 2013)
	3D design of spiked ultraflexible substrates	Rubehn and Stieglitz, 2010; Wang M. et al., 2018
	Neural probe with sinusoidal design and a 3D spheroid tip	Sohal et al., 2014
	Microgeometry and implant thickness effect	Ward et al., 2002
Material morphology	Cationic polymer coatings and PLA and electro-spun fiber meshes with plasma-polymer coating	Ma et al., 2011; Lucke et al., 2018
Surface porosity	Channel size control through (pHEMA-co-MAA) hydrogels	Madden et al., 2010
	PU-based porous implants	Ward et al., 2002
Size and spherical geometry	Alginate spheres/capsules	Veiseh et al., 2015
**Intervention strategy**		
Soft robotics	Control over fluid flow and shear stress through milliscale dynamic soft reservoir with actuatable membrane	Dolan, 2019
Microfabrication	Micro-machined neural prosthetic devices: flexible polymer-based microelectrodes with different shape, size and geometry	Reviewed in Szarowski et al. (2003); Lee et al. (2004), Metz et al. (2004); Polikov et al. (2005), Spataro et al. (2005); Ziegler et al. (2006), Winslow and Tresco (2010); Blau et al. (2011), Gerwig et al. (2012); Gao et al. (2013), Minev et al. (2015); Qi et al. (2017), Vitale et al. (2018); Kozai (2018), Fallahi et al. (2019), and Kumar et al. (2020) (Elyahoodayan et al., 2020)
	Encapsulation technologies of flexible microelectrodes	Reviewed in Ahn et al. (2019)
	Electrically-responsive flexible microfibers	Chen et al., 2017
	Microfabrication of a neural probe with sinusoidal design and a 3D spheroid tip	Sohal et al., 2014
	Wireless, flexible, film-based carbon-polymer composite microelectrode system	Lim et al., 2020
	Additive manufacturing of microelectrode arrays and microneedle arrays	Yang et al., 2016; Morrison et al., 2019; Soltanzadeh et al., 2020
Nanofabrication	CNTs	Reviewed in Aregueta-Robles et al. (2014) (Castagnola et al., 2016)
	Parylene-coated flexible CNTf microelectrodes	Vitale et al., 2018
	Conducting-polymer carbon nanotubes	Abidian et al., 2010; Gerwig et al., 2012; Alba et al., 2015; Mandal et al., 2015; Samba et al., 2015; Du et al., 2018; Altun et al., 2019; Kałuża et al., 2019; Zheng et al., 2019
	PPy nanowires	Reviewed in Qi et al. (2017)
	PPy nanoparticles	Hosseini-Nassab et al., 2017
	SWCNT-PPy/PEGDA composite hydrogels	Xiao et al., 2012
	PPy/CNT films	Luo et al., 2011
	Graphene oxide nanocomposite films of PPy	Weaver et al., 2014
	PLGA nanoparticles embedded in alginate hydrogels	Kim and Martin, 2006
	Nanoparticle-coated nanoelectrodes	Bazard et al., 2017
	Nanoscale biomimetic surfaces	Reviewed in Von Der Mark et al. (2010)

*PEG, polyethylene glycol; DMA, dimethacrylate; PHEMA, poly(2-hydroxyethyl methacrylate); FIB, focused ion beam; PLA, poly(l-lactide-co-d/l-lactide); pHEMA-co-MAA, poly(2-hydroxyethyl methacrylate-co-methacrylic acid); PU, polyurethane; CNTs, carbon nanotubes; CNTf, carbon nanotube fiber; PPy, polypyrrole; SWCNT-PPy/PEGDA, single-walled carbon nanotubes-polypyrrole/poly(ethylene glycol) diacrylate; PLGA, poly(lactic-co-glycolic acid). References: except were specifically indicated as ‘Reviewed in,’ all others are research articles.*

### Developing Innovative and Advanced Functional Biomaterials

Recently, other research groups worked on the development of more suitable materials that can be tolerated by the neural tissue, leveraging on material chemistry, micro- and nano-fabrication techniques (Fekete and Pongrácz, 2017). Many different polymers turned out to be possible substrates of neural interfaces due to their proper flexibility, stability, insulation properties and biocompatibility (Svennersten et al., 2011; Ordonez, 2012; Ware et al., 2013; Nguyen et al., 2014; Arreaga-Salas et al., 2015; Boddupalli et al., 2016). Noteworthy, among these are: shape memory polymers (SMPs) [such as polyurethanes, polylactides, polystyrenes, poly(cyclooctene), thiol-enes and poly(vinyl acetate)]; the widely used micro-electro-mechanical systems (MEMS) polymer materials, namely polyimide, parylene C, PDMS and SU-8 (an epoxy-based photoresist suitable for microelectronic applications). In the soft neural tissue, the use of new smart SMPs is gradually overcoming the one of more stiff materials, as the former seem to drastically reduce the inflammatory response in the surrounding tissue becoming compliant after implantation (Ware et al., 2013; Nguyen et al., 2014; Minev et al., 2015). Likewise, in the PNS the use of flexible polymer materials seems to eliminate the mechanical mismatch of compliance between the implanted electrode and the biological tissue (Blakney et al., 2012; Nguyen et al., 2014).

## MEMS Polymer Materials

### Polyimide

It is a highly resistant and biocompatible polymer, made by imide monomers, among the most widely used substrates for the fabrication of the core of novel neural electrodes with metallic tracks, such as Pt and gold, often coated with different biomaterials for counteracting and delaying the onset of the FBR (Oddo et al., 2016; Delgado-Martínez et al., 2017; Wurth et al., 2017; de la Oliva et al., 2018c). Indeed, among the possible neuroprosthetic applications of this polymer, the group of Navarro X. developed a novel double-aisle electrode to regenerate separated nerve fascicles, made of a double-side thin-film of polyimide (Delgado-Martínez et al., 2017). Although such interface allowed regeneration of nerve branches, it caused FBR in chronic implants. The reaction was indeed similar to that obtained previously with other chronically implanted polyimide intrafascicular electrodes and non-obstructive regenerative electrodes (Lago et al., 2007; Garde et al., 2009), thus affecting the quality of neural signal over time. This common limitation when using polyimide electrodes might be overcome through the functionalization of the polyimide core with advanced biomimicry ultra-low fouling organic or synthetic coatings that can be much more tolerated by the implanted tissue. Toward this direction, diverse efforts have been made to reduce the inflammatory response and electrode encapsulation through new biomimetic solutions. One of these involved the coating with bioresorbable layers of molten saccharose for intracortical insertion in rat models (Hassler et al., 2016). Another option was a superhydrophobic coating from a natural Xanthosoma sagittifolium leaf nanocasted on an electroactive polyimide surface (Chang et al., 2013). A different nanotechnological approach was attempted using hybrid conductive material: an indium tin oxide substrate associated to a nanostructured polyimide film deposited on a glass surface, using a new and simple nanopatterning technique (Rombaut et al., 2019). Very recently, a flexible and transparent polyimide-based electrode was fabricated with a trilayer-stacked geometry that exploits the properties of a high-quality ultrathin film of graphene. This solution showed enhanced power and current efficiencies, with properties comparable to indium tin oxide-based diodes, increased flexibility and long-term stability in different devices (Lee et al., 2019). Finally, another strategy to increase the long-term reliability, while maintaining high flexibility, of a polyimide-based neural interface in free-moving rats, was the one adopted by a research group from China, through a MEMS fabrication approach (Ji et al., 2018). This group developed an innovative optogenetics tool consisting in a polyimide-based hybrid (opto-electric) flexible device that integrates 16 micro-LEDs and 16 IrOx-modified microelectrode arrays. Such device allowed simultaneous, high-resolution optical stimulation and electrical recording of cortical areas. Using this tool, they observed little reduction in the electrical or optical performance for 3 months. Although the fabrication process was quite complex, the device revealed itself to be a promising neural interface for further neuroscience applications, expandable also to larger animals (e.g., non-human primates) and possibly to human patients. However, in order to evade the issue of non-specific protein and cell absorption on the polyimide surface, several groups tried to devise valid alternatives to polyimide substrates, using either diverse MEMS polymers or newly emerged biomedical materials, as shown below.

### Parylene C

Parylene C is a variety of high flexible and chemically inert poly(p-xylylene) polymer commonly used as biocompatible coating and substrate material of electrodes for soft neural implants (Fekete and Pongrácz, 2017). In a recent work, the authors tested parylene C as a substrate material for peripheral nerve interfaces both *in vitro* and *in vivo* (de la Oliva et al., 2018a). In this study, longitudinal devices made of parylene C and polyimide were implanted in the rat sciatic nerve for up to 8 months and the induced FBRs were compared one another. In spite of the advantage to produce parylene C-based thinner substrates than polyimide ones, with no harmful effect on nerve function, long-term stability of such electrodes could be affected by a thicker tissue capsule than polyimide devices. Indeed, the authors observed much more fibroblasts surrounding the former device, thus making parylene C not suitable for chronic implantations (Lecomte et al., 2017; Mueller et al., 2017; de la Oliva et al., 2018a). However, the diverse pattern of FBR around parylene C vs. polyimide, due to their different chemical structures, deserves further investigation before parylene C drops out of other possible invasive neural applications. For example, in another study the authors microfabricated and tested *in vivo* up to 24 months, even though in the rabbit brain, a sinusoidal probe electrode made of a tungsten titanium alloy (WTi) core encased in flexible layers of parylene C with novel design features (Sohal et al., 2014). Interestingly, over the chronic experimental period of the study the electrode performances and neuronal integration were better than other conventional electrodes used for recording of neuronal activity in humans, showing low levels of gliosis. Another interesting attempt to improve the long-term stability *in vivo* of an intrafascicular neural interface (i.e., a flexible microelectrode array with a recording system), was made through a mechanically enhanced flexible interconnection cable using a combination of parylene C and polyimide (Kang et al., 2019). The former provided chemical and electrochemical stability while the latter improved the mechanical strength and handling, with no damage reported, during the implantation procedure of the whole neural interfacing device in canine sciatic nerves. However, before clinical translation, these promising results need more investigation to test their reproducibility in chronic implants of peripheral nerves in larger animal models. Despite the many benefits of parylene C as conformational coating, such as its chemical inertness, there are also significant disadvantages that can limit its wider application compared to the liquid epoxy or silicon coatings. Notably, a better performance and a more controlled deposition process of the latter that are, moreover, much more cost-effective in their production-run make them a preferable choice for researchers. Furthermore, the chemical vapor deposition process required to apply parylene C onto a surface, especially a conductive-metal one, is not only time-consuming but also costly in the attempt to increase its metal adhesion through different methods.

### Poly(Dimethylsiloxane) (PDMS)

This silicon-based organic polymer is the elective material for microfabrication of microfluidic devices including microelectrodes, with tissue-like elastic modulus, easily compliant to neural tissue. These flexible electrodes are usually realized through the process of replica molding, from a master obtained by soft photolithography with a SU-8 photoresist (Qin et al., 2010). Alternatively, they can be fabricated *via* simple and cost-effective photolithography-free methods, such as laser micromachining and master molding of PDMS. Such versatile processes give rise to planar metal electrodes with microfluidic channel geometries (Chatzimichail et al., 2018), and stable neural interfaces (Gao et al., 2013; Minev et al., 2015).

Poly(dimethylsiloxane) micromachining is not only cheap, and easy to realize with high parallelization, but also suitable for the fabrication of long-term neural implants that are able to produce lower inflammatory response than polyimide-based electrodes (Minev et al., 2015). Flexibility and elasticity of PDMS are clearly advantageous features for the fabrication of neural electrodes, as well as in promoting neuronal maturation (Teixeira et al., 2009; Yang and Suo, 2018). Notwithstanding, because of PDMS hydrophobicity, achieving its stable adhesion to hydrated surfaces and materials, such as hydrogels, can be problematic (Yang and Suo, 2018). Furthermore, the proper stability and adhesion between different layers of elastic polymers in implantable electronic devices, such as stretchable electrodes, is difficult to achieve. Actually, under the pressure of muscle contraction and of the strain imposed by the micromotion between nerve tissue and the implant, the electrode can crack. This issue can eventually jeopardize the device functionality. Therefore, alternative solutions have been pursued using all-polymer and metal-free microelectrode arrays with a mixture of various stretchable polymers and *via* replica molding with PDMS (Blau et al., 2011; Guo et al., 2014; Qi et al., 2017), although with mixed fortunes, as described in the next section.

## Other Advanced Biomedical Materials

From the close collaboration between the bioengineering field and the biomedical research area in the development of novel biomaterials for chronic neural applications, diverse strategies are being pursued to decrease the FBR in the next-generation neural interfaces. Some of them are based on the use of organic and synthetic polymeric coatings, including conductive polymers (CPs). Among organic coatings, CPs have been recently investigated with the aim to improve the long-term performance of neural electrodes as they can increase their effective surface, thereby decreasing the impedance, and enhance the electrical properties of neural interfaces, thus seeming the most promising materials (Wilks et al., 2011; Charkhkar et al., 2016). In particular, Poly (3,4-ethylenedioxythiophene) PEDOT, and some of its modified and hybrid versions, have been shown to be safe and reliable candidates in neuroprosthetic applications, being stable and able to improve neural adhesion, electrochemical impedance and dramatically reduce electrical noise and host tissue response (Abidian et al., 2010; Green et al., 2013; Ferlauto et al., 2018; Ganji et al., 2018). Moreover, PEDOT can be easily doped and bio-functionalized with anti-inflammatory drugs, such as dexamethasone (Alba et al., 2015; Boehler et al., 2017; Kleber et al., 2019). It can also be conjugated with other biocompatible and bioinert materials, such as PDMS thin films, CNTs, tetrafluoroborate (TFB), poly(styrenesulfonate), alginate and nafion to guarantee electrochemical stability both *in vivo* and *in vitro* (Blau et al., 2011; Alba et al., 2015; Charkhkar et al., 2016; Ferlauto et al., 2018; Carli et al., 2019). To date, PEDOT functionality has already been demonstrated *in vitro* in terms of improvement of neurite outgrowth bioactivity, and stability of neural micro-stimulation (Green et al., 2009; Mandal et al., 2015). Nonetheless, the long-term performance and integrity *in vivo* of such coatings for chronic recordings have yet to be verified, despite some interesting data collected from short-term epicortical and epidural recordings (Blau et al., 2011). However, these aspects start to be evaluated with promising long-term results, such as for the chronic intracortical neural recordings with high stability and activity in rat motor cortex and mice visual cortex, which deserve further investigation (Charkhkar et al., 2016; Ferlauto et al., 2018; Carli et al., 2019). Another important example was provided by a research team that developed a metal-free electrode array of polypyrrole/polycaprolactone-block-polytetrahydrofuran-block-polycaprolactone (PCTC) sandwiched in between films of PDMS. This group compared the *in vivo* performance of such all-polymer interface with a Pt electrode of the same area in a rat (Guo et al., 2014). They demonstrated a lower impedance of the metal-free device, along with excellent electrical stimulation performances in a stimulated rat hind-limb muscle following squeezing of the sciatic nerve and higher charge injection capacity compared to the Pt electrode, as well as to other PEDOT-coated metal electrodes. Future work from the same group will be necessary to improve and characterize the device physical integrity and mechanical performance in long-term *in vivo* assays also in peripheral nerves.

Two of the most widely used synthetic polymers for coating electrodes are poly (ethylene glycol) PEG (Drury and Mooney, 2003; Gutowski et al., 2015) and PHEMA (Jhaveri et al., 2009; Mario Cheong et al., 2014), as they can form hydrogels with low- or non-fouling characteristics *in vivo*, thus enhancing tissue response around implanted electrodes. However, their long-term use is limited by oxidative mechanisms that partially compromised non-specific protein absorption and device performance. Therefore, recent hybrid solutions have been proposed to overcome some of the issues related to their prolonged stability and sensitivity *in vivo*, such as hybrid thin film photopatternable polymers, combining the properties of PEDOT with the long-term (over 10 days) moisture stability of PEG (Zhu et al., 2017). Another successful test was the integration between PEDOT-poly(styrene sulfonate) (PSS)-CNT nanocomposites and biocompatible PHEMA hydrogels (Castagnola et al., 2016), for potential acute and chronic flexible and high sensitivity electronic applications in rat brains. Thus, the PHEMA hydrogel was able to guarantee the electrochemical performance of the device and improve the quality of intracortical recording until 28 days after the implant, along with the advantage of reducing the mechanical mismatch between neural tissue and device preventing the nanomaterial detachment. Instead, other researchers produced a polydopamine-based coating, resistant to protein adsorption, also for potential applications in intraneural electrodes (Kwon et al., 2016). They developed a polydopamine melanin (PDM) film in the nanometer-scale, a synthetic analog of the two naturally-occurring chemicals dopamine and eumelanin holding unique ionic and electronic properties (Ambrico et al., 2013, 2014; Wünsche et al., 2013), which could be harnessed to increase neural electrodes performance by improving their *in vivo* biocompatibility, while reducing their interfacial impedance. However, further studies will be needed to verify the potentiality of such PDM films. Another group biofunctionalized roughened Pt black (BPt) peripheral nerve cuff electrodes for chronic implantation in animal models using two coatings of PEG or nafion, with the latter showing low interfacial impedance, together with good stability and reduced fibrotic capsule, thus justifying deeper investigation also for possible clinical applications (Lee et al., 2017). A different research team developed a novel CP for neural electrodes made of a soft wire conductive matrix, which showed optimal mechanical (suitable flexibility) and electrochemical properties, as well as excellent biocompatibility after 1 month implantation in a rat sciatic nerve (Zheng et al., 2019). The conducting core of the electrode was based on silicone/poly(3,4-ethylenedioxythiophene)-polyethylene glycol (PEDOT-PEG) elastomer encapsulating 3D CNTs, and it was shown to be more compliant to soft nerve tissue than traditional polyimide implants in terms of FBR. Finally, another CP frequently used as electromechanically active coating for biosensors, implantable gold electrodes (Yamato et al., 1995; Cui et al., 2003; Green et al., 2008), fiber scaffolds capable of dynamic mechanical actuation (Gelmi et al., 2016) and microelectrode arrays (Qi et al., 2017; Du et al., 2018), is the polypyrrole (PPy). However, it has often shown limited performances and chronic recording failure over extended periods of time *in vivo*, also due to chronic inflammation and fibrotic encapsulation (Yamato et al., 1995; Cui et al., 2003; McConnell et al., 2009). In a recent work, a research group tried to improve the performance of biosensing interfaces based on copolymerization of benzenamine-2,5-di(thienyl)pyrrole (SNS-An) with 3,4-ethylenedioxythiophene (EDOT) (Altun et al., 2019). The so-developed copolymer films showed increased biosensing efficiency after the incorporation of CNTs and fullerene, albeit evidence of the effect of such copolymerization on their performance *in vivo* is still missing. Conversely, others observed high conductivity and good performances in their *in vivo* recordings of rat electrocorticographic signals, and in the stimulation of the sciatic nerve of the animals through the use of stretchable polymeric microelectrode arrays. These arrays were composed of PPy electrodes anchored to an underlying PDMS film using PPy nanowires. Moreover, these flexible devices showed high stretchability with no cracking, high resistance up to 100% strain and good electrode-substrate adhesion (Qi et al., 2017). To sum up, composite PEDOT-PEG or PEDOT-PHEMA solutions would seem to offer a suitable compromise between long-term mechanical and bio-stability as well as high electrical performance ensuring, at the same time, very good biocompatibility, if were not for the current limit of the few available *in vivo* results against FBR.

## Hydrogels

The use of highly hydrated and ultralow-fouling polymeric hydrogels outperforms other coating materials in terms of biocompatibility although the existing issue of the low electrical properties of some chemical hydrogel compositions. This drawback could be solved by including in hydrogels some of the conductive components examined above, such as CPs and CNTs (Green et al., 2012; Xiao et al., 2012). Another alternative solution could be the use of zwitterionic hydrogels with ionic conductive capacity as well as biomimetic and anti-inflammatory features, which can also resist the FBR for longer time-scale than other synthetic HEMA hydrogels (Zhang et al., 2013; Diao et al., 2019). For instance, in one of these most recent papers, it was demonstrated that highly stretchable, tough and flexible PVA/P(AM-co-SBMA) zwitterionic hydrogels possess high intrinsic ionic conductivity due to the zwitterionic counterions, and could therefore fulfill flexible electrical device applications (Diao et al., 2019). Further examples are represented by the synthesis of ultralow-fouling zwitterionic hydrogels and non-leaching polymeric sulfobetaine (polySB) coatings for subcutaneous implantation of medical devices in animal models up to 2—3 months (Smith et al., 2012; Zhang et al., 2013; Yesilyurt et al., 2017). Another recent paper showed the synthesis and *in vitro* validation of poly(carboxybetaine) zwitterionic hydrogel coating, with a Young’s modulus in the range of the neural tissue, of a polyimide-based device to minimize the fibroblast and macrophage adhesion (Trel’Ová et al., 2019). Similarly, a previous carboxybetaine methacrylate zwitterionic hydrogel synthesized *via* photopolymerization, rather than thermal polymerization, with a more reactive and functionalizable crosslinker showed superior stability at diverse pH values and improved mechanical properties than many other photopolymerized hydrogels (Carr et al., 2011). Finally, in their work some researchers developed a well-controllable electrochemically-mediated surface-initiated atom transfer radical polymerization (e-siATRP) method to fabricate a superlow protein absorption zwitterionic hydrogel coating that was based on poly(sulfobetaine methacrylate) (pSBMA) (Hu et al., 2015). The main advantage of the present method is represented by the usage of the commercially available SBMA and its very easy and controllable synthesis process, which can be also applied to implantable neural electrodes with optimal biocompatibility and antifouling capacity as proven by *in vitro* tests (Hu et al., 2015).

Besides, another frequently encountered issue related to such systems is the delamination of the hydrogel from the electrode surface, and thus the establishment of adequate patterning methods for binding it to the substrate. In a recent work, microsystem engineers and chemists addressed these problems by developing a new hybrid conductive system made from the combination between the synthetic hydrogel P(DMAA-co-5%MABP-co-2,5%SSNa) and the conducting polymer PEDOT, which can be covalently attached to the electrode surface and patterned using a photolithographic process *via* UV irradiation. In such a way, the authors created an interpenetrating network, suitable for coating neural microelectrodes, showing excellent electrochemical stability and no toxicity *in vitro* (Kleber et al., 2017). Conductive hydrogel coatings can ameliorate the electrical properties and performances of conventional metal electrodes, with lower energy demand to interface with and control target nerve activity. To achieve a suitable response from a distant stimulated nerve, the application of higher currents is necessary with possible adverse reactions, such as the corrosion of the uncoated metallic electrode and its failure over time. Hence, due to their high efficiency and electrochemical stability, conductive hydrogels can provide stable and long-term activity also when applied to stainless steel (SS) electrode arrays in peripheral nerves as showed in this work (Staples et al., 2018). The researchers fabricated planar electrode arrays by electrodepositing a thin layer of PEDOT/pTS onto the SS electrode and then coating it with a 20 wt% poly(vinylalcohol)-methacrylate-taurine (PVA-taurine) hydrogel. In their *in vitro* tests the conductive hydrogel coating improved electrochemical properties and device stability over 42 days regardless of the underlying metallic substrate of the electrode. Nonetheless, the authors used non-penetrating cuff-electrodes and only for *in vitro* analysis, thereby the benefit of such hydrogel coating against FBR over chronic invasive implant periods *in vivo* will be the focus of their future work. Accordingly, their principal task will be the demonstration of low scar tissue development due to the reduced hydrogel stiffness and to its natural anti-fouling properties.

Modulation of the FBR for intraneural interfaces can also be achieved taking inspiration from recent works in animal models of type-I diabetes (Vegas et al., 2016; Bochenek et al., 2018). In these *in vivo* studies the authors performed encapsulation of human pancreatic β-cells with chemically modified alginate formulations [i.e., triazole-thiomorpholine dioxide (TMTD) alginate, Z2-Y12 and Z1-Y15 immune-modulating alginate derivatives] to long-term protect cells from the chronic response of the immune system, without the need for broad immunosuppression. In particular, these different hydrogel formulations increased the immunoprotection of cells in immune competent mice and non-human primate models, successfully reducing FBR and preventing from pericapsular fibrotic overgrowth. Similar strategies with alginate hydrogels could therefore be translated into clinical practice to encapsulate intraneural electrodes, and exploited to overcome the challenge of foreign body rejection from the host immune system. Overall, despite the many advantages provided by conductive hydrogel coatings in terms of high electrochemical performance of the device, especially when using zwitterionic formulations, augmented quality of signal recording, reduction of the mechanical mismatch along with ultralow-fouling properties, their long-term stability and functionality *in vivo* still represent main limitations that need to be solved in the next future. In fact, because of their soft texture, highly hydrated jelly structure and low mechanical strength, hydrogels can be slowly degraded or damaged already during the implantation surgery, thus impairing their permanence and performance within neural tissue. However, to the best of our knowledge, at present they are by far the most promising biomimetic coatings in this context.

A summary of the intervention strategies based on the development of advanced functional biomaterials with representative examples is reported in Table 2.

**TABLE 2:** Intervention strategies based on the development of advanced functional biomaterials.

(ii) Advanced functional biomaterials		

Intervention strategy	Examples	References
Novel flexible and biocompatible polymers	Extended overview	Reviewed in Ordonez (2012); Ware et al. (2013), Boddupalli et al. (2016), and Fekete and Pongrácz (2017)
	Polypyrrole microactuators	Svennersten et al., 2011
	Hydrogel core of bacterial cellulose and conductive polymer shell layer of PEDOT	Chen et al., 2017
	PEG-RGD hydrogels	Blakney et al., 2012
SMPs	Extended overview	Reviewed in Ware et al. (2013)
	Bioinspired cellulose nanocomposites	Nguyen et al., 2014
	Thiol-ene based softening substrates	Arreaga-Salas et al., 2015
Micro-electro-mechanical systems (MEMS) polymer materials	Polyimide	Reviewed in Kozai (2018) (Lago et al., 2007; Garde et al., 2009; Mercanzini et al., 2010; Chang et al., 2013; Hassler et al., 2016; Oddo et al., 2016; Boehler et al., 2017; Delgado-Martínez et al., 2017; Wurth et al., 2017; de la Oliva et al., 2018b, c; Ji et al., 2018; Kang et al., 2019; Lee et al., 2019; Rombaut et al., 2019)
	Parylene C	Reviewed in Fekete and Pongrácz (2017) (Ziegler et al., 2006; Sohal et al., 2014; Xie et al., 2014; Lecomte et al., 2017; Mueller et al., 2017; de la Oliva et al., 2018a, b; Vitale et al., 2018; Kang et al., 2019)
	PDMS	Blau et al., 2011; Gao et al., 2013; Guo et al., 2014; Minev et al., 2015; Chatzimichail et al., 2018; Kumar et al., 2020
CP coatings	Extended overview	Reviewed in Aregueta-Robles et al. (2014) and Balint et al. (2014)
Hydrogels	Alginate hydrogels	Reviewed in Lee and Mooney (2012)
	PEG-containing hydrogels	Spencer et al., 2017; Zhang et al., 2017
	PEG-maleimide hydrogel coatings	Gutowski et al., 2015
	Poly(SB) hydrogels	Smith et al., 2012
	PEDOT:PSS/alginate conductive hydrogels	Ferlauto et al., 2018
	Conducting PEDOT/PDMAAp hydrogels	Kleber et al., 2017, 2019
	PHEMA hydrogels	Jhaveri et al., 2009; Castagnola et al., 2016; Zhang et al., 2017
	Conducting hydrogels with biomolecules	Reviewed in Aregueta-Robles et al. (2014) and Lotti et al. (2017) (Green et al., 2012; Mario Cheong et al., 2014; Chen et al., 2017; Staples et al., 2018)
	SWNT-PPy/PEGDA composite hydrogels	Xiao et al., 2012
	Chemically-modified alginate microspheres	Vegas et al., 2016; Bochenek et al., 2018
Zwitterionic hydrogels	Phosphorylcholine polymer	Yesilyurt et al., 2017
	PVA/P(AM-co-SBMA) polyelectrolyte	Diao et al., 2019
	Poly(carboxybetaine) and pCBMA	Jiang and Cao, 2010; Carr et al., 2011; Zhang et al., 2013; Trel’Ová et al., 2019
	Phosphorylcholine self-assembled monolayers	Chen et al., 2005
	Poly(sulfobetaine) and pSBMA	Reviewed in Sin et al. (2014a) (Jiang and Cao, 2010; Sin et al., 2014b; Hu et al., 2015; Wu et al., 2018)
	Zwitterionic hydrogels with bioactive materials	Reviewed in Von Der Mark et al. (2010)

*SMPs, shape memory polymers; RGD, Arg-Gly-Asp motif; MEMs, micro-electro-mechanical systems; PDMS, poly(dimethylsiloxane); CPs, conductive polymers; PEG, polyethylene glycol; Poly(SB), polymeric sulfobetaine; PEDOT:PSS, poly(3,4-ethylenedioxythiophene):Polystyrene sulfonate; PEDOT/PDMAAp, poly(3,4-ethylenedioxythiophene)/poly(dimethylacrylamide-co-4-methacryloyloxy benzophenone-co-4-styrenesulfonate; PHEMA: poly(2-hydroxyethyl methacrylate); SWNT-PPy/PEGDA, single-walled carbon nanotubes-polypyrrole/poly(ethylene glycol) diacrylate; PVA/P(AM-co-SBMA), polyvinyl alcohol/acrylamide and sulfobetaine methacrylate copolymer; pCBMA, poly(carboxybetaine methacrylate); pSBMA, poly(sulfobetaine methacrylate). References: except were specifically indicated as ‘Reviewed in,’ all others are research articles.*

### Interface–Microenvironment Interaction

The aqueous characteristic of synthetic and organic hydrogel coatings, such as PEG-based and zwitterionic-based formulations, and their synthesis methods could be harnessed for therapeutic purposes. In order to modulate locally the immune response of the host tissue, various hydrogel formulations could represent a means to encapsulate or covalently incorporate growth factors, therapeutic anti-inflammatory and anti-fibrotic medications as well as small-molecule drugs (Jhaveri et al., 2009; Mario Cheong et al., 2014; Gutowski et al., 2015; Doloff et al., 2017). To this aim, a considerable list of potential therapeutic drugs could be loaded during polymer fabrication into biodegradable CPs, polymeric coatings and hydrogels (Lotti et al., 2017; Zeglio et al., 2019), and many others could be tested as good candidates for contrasting FBR. In the following sections we will take into account some of the most promising lead compounds and novel drug delivery strategies to further improve the biological response to the electrodes in chronically implanted nerve tissues.

## Dexamethasone

One of the most frequently anti-inflammatory agents loaded into electrode coatings for chronic applications is the corticosteroid drug dexamethasone and its phosphate derivative (Spataro et al., 2005; Kim and Martin, 2006; Mercanzini et al., 2010; Alba et al., 2015). Interestingly, in two FBR models in the rat sciatic nerve, one with longitudinal parylene C intraneural implants, and the other with longitudinal polyimide-based implants, the beneficial effects of dexamethasone were clearly demonstrated (de la Oliva et al., 2018b). In fact, in this work only subcutaneous administration of dexamethasone up to 8 weeks, compared to other anti-inflammatory drugs (i.e., ibuprofen, maraviroc, and clodronate liposomes), was able to reduce the inflammatory reaction as well as matrix deposition around the electrodes in a comparable manner. In another model of FBR, developed by the same group, using TIME interfaces implanted in the rat sciatic nerve, the long-term functionality (i.e., 3 months) of the electrodes was maintained by systemic administration of dexamethasone. The drug was indeed able to reduce the loss of functioning contacts of the TIMEs that stimulated the target nerves and evoked a muscle response while reducing the inflammatory cell infiltration during the first month, which is the critical time-frame for FBR development (de la Oliva et al., 2019). Since dexamethasone showed similar beneficial effects in different devices and substrates, it may represent an ideal drug treatment to extend the implant functionality over time in peripheral nerves. Accordingly, the use of dexamethasone could be combined with tissue engineering strategies, such as substrate functionalization with biodegradable hydrogels and porous CPs, for its controlled local release in order to specifically target its activity around the implant, while reducing potential side effects caused by its systemic toxicity at too high doses. In relation to this approach, one of the first *in vitro* attempts to control the release of dexamethasone from a conducting polymer coating of PPy on Au electrode sites was done through an electrochemically-controlled release of dexamethasone phosphate as a dopant (Wadhwa et al., 2006). The authors elicited an anti-inflammatory response in murine glial cells, although they experienced a low adhesion of the coating to the electrode, turning out to be unable to sustain an extended drug-delivery time. Instead, MWCNT and dexamethasone-doped electropolymerized PEDOT coatings have shown promise to improve chronic neural electrode performance. Indeed, despite the impedance increase, coated electrodes successfully recorded neural activity throughout the implantation period (Alba et al., 2015), and showed excellent stability and no signs of inflammation, in response to electrical stimulation, over 45 days in rat brain. Similarly, another team filled MWCNTs with a solution of dexamethasone phosphate and then sealed the open ends of the nanotubes with a film of PPy, *via* electropolymerization, as electrode coating for an on-demand drug release strategy (Luo et al., 2011). The researchers detected an effective anti-inflammatory activity *in vitro*, and the smaller the size of the nanotubes the higher the drug release. Furthermore, such PPy coating significantly decreased the electrode impedance. However, despite some preliminary evidence of the dexamethasone success, there are still a few reliable data *in vivo* and some considerable kinks to work out before long-term use of the drug as a resolutive anti-inflammatory treatment for clinical applications in humans. For instance, an important issue, not only related to dexamethasone but to any other loaded chemicals, is that of the drug exhaustion around the implant microenvironment.

## Anti-Fibrotic Drugs

It has recently been found another molecular target underpinning the development of the FBR. Actually, targeting colony stimulating factor-1 receptor (CSF1R), which is upregulated on the macrophage surface after implantation of different biomaterials, including biocompatible hydrogels, may represent a smart strategy to hamper fibrosis and capsule formation (Doloff et al., 2017). Such therapeutic approach may indeed avoid to directly targeting macrophages or applying massive immunosuppression with possible harmful side effects to the whole organism.

Another potential target protein is the connective tissue growth factor (CTGF), a key player underlying the progression of the fibrotic reaction driven by TGF-β, which is quickly induced by TGF-β in different contexts of fibrotic disease as a specific downstream effector of its activity (Leask et al., 2002). To date, the *in vivo* silencing of target genes involved also in chronic disease such as fibrosis, including CTGF, can be achieved through various therapeutic strategies, either *via* local or by means of systemic administration of viral and non-viral vectors. One of the most promising strategy is represented by the gene therapy through the selective gene knock-down mediated by the small interfering RNAs (siRNAs) or the microRNAs (miRNAs) (Lam et al., 2015; Salazar-Montes et al., 2015; Omar et al., 2016). These therapeutic molecules are short non-coding RNAs with a great potential for different clinical applications (Gori et al., 2015; Lam et al., 2015). However, in order to increase the silencing efficiency of siRNAs and miRNAs the search for the most suitable carrier in terms of low toxicity and immunogenicity to target cells remains an open challenge. In such a scenario, NP-based delivery of siRNAs might represent an ideal solution by improving not only the safety of this potential therapy, but also its effectiveness (Surendran et al., 2017; Yu et al., 2020). The main advantages of NPs are their tunable size, shape and surface features along with their adjustable biological properties (Miele et al., 2012). Among the various material formulations tested, including gold, silica, porous silicon, CNT and diverse polymers, magnetic iron oxide NPs seem to be the most interesting for gene therapy due to their reduced toxicity, easy surface modification and high versatility in a wide range of biomedical applications (Wu et al., 2008; Xiao et al., 2014; Saeed et al., 2018). In this regard, a research group has recently investigated *in vitro* the anti-fibrotic activity of polyethyleneimine (PEI)-functionalized magnetic iron oxide NP-mediated delivery of siRNAs against CTGF (Yu et al., 2020). The siRNA-loaded NPs showed low cytotoxicity and high transfection efficiency, along with significant CTGF silencing performance, reducing collagen production and deposition in the hepatic stellate cell line LX-2. Thus, taking the cue from this study one could envision the use of invasive electrodes with nanoparticle-embedded coatings, such as hydrogels, to regulate the controlled delivery of siRNAs or miRNAs for the specific silencing of CTGF, or other mediators of inflammation and fibrosis.

## Further Tissue Engineering Strategies for Targeted Drug Release

Ideally, drug loading within the coating of an implantable neural device with tunable physicochemical characteristics, can help avoid adverse side effects associated to systemic administration thanks to the controlled local delivery of the appropriate amount of the drug and for the desired time-window. In the last decades, a considerable number of brilliant approaches have been attempted in order to dope, absorb and incorporate in the interface coating the desired drug to accomplish a safe, effective, controlled and long-term pharmacological release as the aforementioned examples with dexamethasone. Drug loading into the coating can be realized through a self-assembly procedure, by means of electrostatic interactions, using a charged drug as dopant agent or hydrophobic interactions, *via* physical entrapment, as well as covalent bonding using degradable peptides or cleavable molecular linkers (Balint et al., 2014; Zeglio et al., 2019). The miniaturization of biomedical devices through the use of microfluidics is a novel opportunity for tuning the properties of flexible and stretchable biomaterials in many smart applications, from biology to medicine and tissue engineering, including drug delivery purposes (Fallahi et al., 2019). Also, novel technologies for long-term encapsulation using new arising materials have recently emerged with promising results. These include: thin-films of inorganic coatings of Al_2_O_3_ (alumina), SiO_2_ (silica), SiC (carborundum) and diamond; in addition, several organic coatings have been used, which are made of – among others - parylene C, polyimide, liquid crystal polymer (LCP), SU-8 and silicone elastomer for implantable microfabricated medical devices. Both chemical solutions, organic and inorganic, leverage on the miniaturization of the implants thanks to the material flexibility and scalability (Ahn et al., 2019). Although the extended suitability - over several decades – of these materials for the encapsulation of biomedical devices has been largely demonstrated in the literature, they have not yet been approved for chronic implantation in patients (Ahn et al., 2019). However, for the prospective chronic encapsulation of microfabricated implants, these novel materials could overcome the performances of conventional macroscale ceramics- or metal-based packaging technologies that offer scarce adaptability to the microfabrication processes; these emerging materials possess indeed largely tunable physicochemical properties and higher biocompatibility as well as reliability for clinical applications in order to control the FBR. Nevertheless, much efforts need to be done for their complete processing and engineering, in particular a multi-combinational approach with the association of various organic and inorganic materials will be advisable in the next future for developing an optimal deposition process and studying their barrier characteristics (Ahn et al., 2019). In a representative example of such approach, the authors compared the long-term behavior of Utah electrode array-based neural interfaces, encapsulated in a bilayer of Al_2_O_3_ and parylene C, vs. the same electrodes with parylene C-only encapsulation (Xie et al., 2014). They observed higher performance stability (i.e., stable power-up frequencies and constant radio-frequency signal strength) and thus increased lifetime of the bilayer encapsulated devices compared with the parylene-only devices. Moreover, the former represented a more reliable encapsulation method for the functionality of chronically implanted neural interfaces.

As regards the investigation of ideal biomaterials for controlled drug delivery, in an *in vitro* analysis three different types of carefully designed pHEMA hydrogel coatings were applied to microfabricated neuroprosthetic devices, through specific hydrogel casting methods, with incorporation of lysine and NaCl to increase both storage capacity and local pharmacological delivery rate in the brain (Jhaveri et al., 2009). Although promising in terms of favorable neural cell response upon Nerve Growth Factor (NGF) delivery, their study needs to be refined for extended *in vivo* applications. In fact, the mechanical properties of these coatings need to be improved to mimic more closely those of peripheral nervous tissue in order to avoid delamination following their insertion in the body; also, more detailed studies should be planned for determining specific local drug delivery and degradation time *in vivo* besides the NGF tested herein. In another interesting *in vitro* investigation on new smart multifunctional biomaterials, electrically-responsive core-shell hybrid microfibers, coated with PEDOT by chemical polymerization, were used for the controlled release of the anti-inflammatory diclofenac sodium salt (Chen et al., 2017). The microfibers were fabricated through a combination of co-axial wet spinning of a hydrogel core of bacterial cellulose, using a microfluidic device, and a dip-coating method of the hydrogel with a conductive polymer shell layer of PEDOT. The developed hybrid microfibers showed very high biocompatibility, electroactivity and allowed the researchers to control the diclofenac release *via* external electrical stimulation in a rat neural cell line. Instead, a remarkable intervention strategy at relevant time scales for chronic clinical applications was the one proposed by Boehler et al. (2017). They microfabricated flexible layers of polyimide on a Pt-IrOx electrode with subsequent coating of PEDOT to harness its conductive properties and drug delivery capacity, for sustained (12 weeks) dexamethasone delivery in implanted rat brains. The drug was incorporated during the polymerization step of PEDOT and released in a controlled manner for attenuating the FBR over the 12-week period that is way beyond the initial healing phase of 6 weeks. Instead, an engineered PEG-maleimide hydrogel coating for neural electrodes was developed to actively control the local release of an anti-inflammatory molecule (IL-1Ra) *in vivo* (Gutowski et al., 2015). They tuned the physiochemical properties of the hydrogel by developing a stimulus-responsive degradable portion for on-demand release of the anti-inflammatory agent in rat brain tissue. Indeed, by taking advantage of the high expression of matrix metalloproteinases (MMPs) in the inflamed rat brain, the authors functionalized the hydrogel coating with MMP-degradable crosslinking peptides that were able to release IL-1Ra at the brain-implant interface in response to inflammation. Altogether, they observed only a moderate reduction of inflammatory markers, although neuronal survival around the electrodes was higher than uncoated controls. However, further improvements are necessary to verify the efficacy of this strategy also in peripheral nerves and for chronic implants, such as a reduced adhesion to the coating of other cell types besides glial and neuronal cells. Importantly, since no evident differences were detected in the recruitment and activation of inflammatory cells involved in scar formation between coated and uncoated implants, it will be of utmost importance to work more on this aspect. Another interesting avenue leverages the properties of conducting and conjugated polymer-based devices to create a drug-eluting electrode. The device can be loaded with the drug of choice and the release is electronically triggered by electrostatic interactions and/or electrical stimulation. In one of these studies, the drug of interest was entrapped in an electropolymerized PEDOT:PSS film by means of a gentle supercritical carbon dioxide (scCO_2_) treatment and then gradually released *in vitro via* electrical stimulation, retaining an elevated activity while maintaining normal electrochemical properties of the polymer surface (Löffler et al., 2016). Such scCO_2_-based method could represent a smart approach for loading anionic and cationic drugs in any conductive bio-coating, which can be adjusted depending on the purpose. A different strategy that exploited the mixed conductivity of PEDOT:PSS is the one based on implantable ion pumps (Isaksson et al., 2007). Such technological platform can be potentially utilized not only for targeted ion delivery, but also for larger biomolecules, such as glutamate, aspartate and y-aminobutyric acid (GABA) (Simon et al., 2009), which can be particularly useful for the treatment of CNS disorders. For example, a clinically relevant *in vivo* application of an ion pump for controlled GABA delivery was carried out in rat models of peripheral nerve injury (Jonsson et al., 2015). In this study, a specific design of the outlets of the implantable organic electronic delivery device was developed for local GABA release along the spinal cord. It showed the ability to mitigate neuropathic pain with no side effects.

A recent advancement of this technology for *in vivo* applications, was the development of a microfluidic ion pump with high drug-delivery ability (Uguz et al., 2017). The major advantage of this novel configuration was represented by the reduced distance for the electrophoretic transport of the drug, requiring a low voltage for its delivery, and by the fact that the microfluidic channels were connected to an almost inexhaustible drug reservoir. A similar microfluidic ion device with PEDOT:PSS-based recording electrodes was fabricated for releasing GABA in a specific brain region of an epilepsy mouse model (Proctor et al., 2018). The device, composed by a neural probe incorporating a microfluidic ion pump and neural electrodes for recording neural activity, allowed the inhibitory neurotransmitter to be selectively delivered to the seizure source for its control and termination. Accordingly, these ion pump devices may represent very interesting spatially and temporally controlled electrophoretic drug delivery systems. These implanted platforms may hold tremendous potential for on-demand therapeutic drug delivery also when combined to intraneural electrodes. The authors speculate that their device could be useful also in chronic drug delivery settings for reducing the FBR after additional technological developments, especially related to the drug reservoir reloading and the improvement of their long-term biostability.

Other investigators generated graphene oxide (GO) nanocomposite films, deposited inside a conducting PPy scaffold, to enhance the dexamethasone phosphate-loading capacity of the graphene component, by means of physical adsorption, and to elicit its electrically controlled delivery (Weaver et al., 2014). *In vitro* tests carried out in primary rat astrocytes showed the possibility to finely control and adjust the drug-release time and dosage, depending on the need, by varying the ultrasonication time required to prepare the graphene oxide nanosheets. Thereby, modulating the ultrasonication treatment one can influence the film morphology, drug load and release profile in a versatile manner. However, many of these smart tissue engineering strategies have failed to reach patients’ bed because of a series of drawbacks. Among them, it is worth considering the frequent lack of a suitably charged dopant molecule and the poor drug loading performance with low and unsatisfactory concentration, particularly when using conjugated and conductive polymeric films. Besides, additional hurdles to be addressed include film instability and drug leakage. In particular, the undesired leakage of the drug from the polymeric coating may indeed occur when the loaded molecule is too small compared to the pore size of the releasing hydrogel (Zeglio et al., 2019). As for the limitation of poor drug loading performance, mostly for delivering large molecule therapeutics, it can be addressed through the use of NPs made of conductive polymers (such as PPy) (Hosseini-Nassab et al., 2017). Thanks to their higher surface area than conventional conductive thin films, the electroresponsive PPy NPs enabled a controlled and efficient release of surface-loaded bioactive insulin, triggered by electrical stimuli on a coated Pt electrode, also in *in vivo* tests of therapeutic delivery in mouse models. The authors speculate that such drug-loaded NPs may be enclosed into a semi-permeable hydrogel coating that allows only drug molecules to pass through. In conclusion, this strategy could be potentially envisioned also for peripheral neural interfaces. Such implantable drug delivery system could improve spatially and temporally controlled drug release by simply varying the ratio of the quantity of NPs to the concentration of the desired drug, while maintaining its bioactivity.

Lastly, alternative and smart methods for interfering with the interaction between device surface and tissue microenvironment, thereby evading the host immune response, could be represented by: (a) modifications of biomaterial surface with adhesive peptides (e.g., RGD cell adhesion ligand on PEG surfaces) to partially attenuate inflammatory reaction and capsule formation (Lynn et al., 2011); (b) functionalization of PEG-coated surfaces with synthetic human-based “self” peptides (e.g., the immunomodulatory membrane proteins CD47 and CD200) to inhibit macrophage-mediated clearance of the surface and prolong its *in vivo* survival (Rodriguez et al., 2013; Kim et al., 2014).

A summary of the intervention strategies based on the control of the interface-microenvironment interaction with representative examples is reported in Table 3.

**TABLE 3:** Intervention strategies based on the control of the interface-microenvironment interaction.

(iii) Interface-microenvironment interaction		

Intervention strategy	Examples	References
Targeted drug delivery systems	Extended overview	Reviewed in Cai and Xu (2011)
Anti-inflammatory drugs	Dexamethasone	Reviewed in Lotti et al. (2017) and Zeglio et al. (2019) (Spataro et al., 2005; Kim and Martin, 2006; Wadhwa et al., 2006; Mercanzini et al., 2010; Luo et al., 2011; Weaver et al., 2014; Alba et al., 2015; Boehler et al., 2017; de la Oliva et al., 2018b, 2019; Kleber et al., 2019)
	IL-1Ra	Gutowski et al., 2015
	Ibuprofen	de la Oliva et al., 2018b
x	Clodronate liposomes	de la Oliva et al., 2018b
	Diclofenac	Reviewed in Zeglio et al. (2019) (Chen et al., 2017)
	RGD cell adhesion ligands on glass and PEG surfaces	Reviewed in Zeglio et al. (2019) (Anderson et al., 1999; Lynn et al., 2011; Blakney et al., 2012)
	Functionalization of PEG surfaces with human self-peptides	Reviewed in Lotti et al. (2017) (Kim et al., 2014)
Anti-fibrotic drugs	Extended overview	Reviewed in Lotti et al. (2017)
	Targeted silencing of CTGF via siRNAs-, miRNAs- and nanoparticle-based silencing	Reviewed in Leask et al. (2002); Wu et al. (2008), Miele et al. (2012); Gori et al. (2015), Salazar-Montes et al. (2015); Omar et al. (2016), Surendran et al. (2017), and Saeed et al. (2018) (Xiao et al., 2014; Yu et al., 2020)
	CSF1R inhibition	Doloff et al., 2017
Tissue engineering strategies for targeted drug release	Extended overview	Reviewed in Ratner (2002); Drury and Mooney (2003), Knop et al. (2010); Balint et al. (2014), Gori et al. (2015); Lam et al. (2015), Salazar-Montes et al. (2015), and Zeglio et al. (2019)
	Human self-peptides	Rodriguez et al., 2013
	Conductive polymer films	Wadhwa et al., 2006; Mario Cheong et al., 2014; Löffler et al., 2016
	Electrically-responsive microfibers	Chen et al., 2017
	Milliscale dynamic soft reservoir (DSR)	Dolan, 2019
	Embedded microfluidic channels	Metz et al., 2004; Retterer et al., 2004; Ziegler et al., 2006; Gao et al., 2013; Takehara et al., 2014; Minev et al., 2015; Elyahoodayan et al., 2020
	Hydrogel coating (e.g., pHEMA, PEG-maleimide, PVA-heparin)	Jhaveri et al., 2009; Mario Cheong et al., 2014; Gutowski et al., 2015
	CNTs nanoreserviors	Luo et al., 2011
	Electronic ion pumps	Isaksson et al., 2007; Simon et al., 2009; Jonsson et al., 2015; Uguz et al., 2017; Proctor et al., 2018
	Microencapsulation	Campioni et al., 1998
	Nanoparticle-based delivery	Reviewed in Wu et al. (2008); Cai and Xu (2011), Miele et al. (2012); Surendran et al. (2017), and Saeed et al. (2018) (Kim and Martin, 2006; Xiao et al., 2014; Hosseini-Nassab et al., 2017; Yu et al., 2020)
	Electrically controlled drug delivery from graphene oxide nanocomposite film of PPy	Weaver et al., 2014

*IL-1Ra, interleukin-1 receptor antagonist; RGD, Arg-Gly-Asp motif; PEG, polyethylene glycol; CTGF, connective tissue growth factor; siRNAs, small interfering RNAs; miRNAs, microRNAs; CSF1R, colony stimulating factor-1 receptor; DSR, dynamic soft reservoir; pHEMA, poly(2-hydroxyethyl methacrylate); PVA, polyvinyl alcohol; CNTs, carbon nanotubes; PPy, polypyrrole. References: except were specifically indicated as ‘Reviewed in,’ all others are research articles.*

## Final Remarks and Future Directions

To date, the design of resolutive solutions to modulate the FBR, based on the exhaustive comprehension of its molecular mechanisms, represents a major challenge for a suitable and long-lasting implantation of intraneural devices.

Modern neuroprostheses may employ electrodes produced with microtechnology that, however, do not go below the size of some tens of micrometers. Various techniques of micromachining and micromolding of flexible and conductive polymer coatings may allow scientists to fine tuning the features of the electrodes to the characteristics of the host tissue, thus creating more stable devices over time. Furthermore, the integration of microfluidic ion pumps and channels into neural probes can be harnessed for extended drug delivery in the implanted tissue, so to dramatically reduce the FBR and to be much better tolerated than plain implants.

Indeed, microfluidics and, most of all, nanofluidics, although promising are still quite unexplored in neuroprosthetics, and deserve further investigation.

Strategies based on microfluidic, microscale and nanoscale technologies provide scalability. They can lead from the long-term and stable neurotransmission simultaneously to many tissue points, to an enhancement of spatial selectivity stimulation, through implantable microelectrode arrays and microscale actuators (Kozai, 2018; Kumar et al., 2020). Even more so because conventional electrodes and recording systems are bulky and unsuitable for single cell resolution. By micro- and nano-engineering the surface properties of the implant, one can obtain a better control of therapeutic drug release from artificial nanopores, NPs made of conductive polymers and other nanostructured materials. Compared to traditional bulk materials, this latter mechanism can take advantage of the higher surface area of loaded NPs, their variable degradation rate depending on the biomaterial used, and the adjustable selectivity and permeability of hydrogel coatings to drug molecules. Biofunctionalization of NPs with antigen-recognized antibodies may further ameliorate targeting efficiency by increasing the drug concentration within a specific tissue (Cai and Xu, 2011).

For example, bioinspired cellulose nanocomposites have higher versatility and functionality than rigid silicone implants, due to their switchable stiffness characteristics, reducing neuroinflammation in chronic implants (Nguyen et al., 2014). Indeed, these mechanically adaptive nanomaterials although initially rigid become compliant after intracortical implantation in rats. They have been proven to lower neuroinflammatory response at chronic time-points, with no neuronal loss, limited scarring, reduced blood-brain barrier damage as well as decreased accumulation of activated microglia and macrophages at the implant-tissue interface. Upon insertion in the brain, when exposed to physiological conditions, the nanocomposites exhibited a massive reduction in tensile storage modulus and, in turn, the induced tissue strain was dramatically lowered (Nguyen et al., 2014).

Moreover, nanofiber-formed nanogels and self-assembly nanoscaffold hydrogels are broadly adopted for targeted and controlled drug delivery. For instance, some antibody-drug conjugate payloads can be maintained in a target area by side chains, chemical moieties and interactions with the nanogel, thus prolonging their protective effect (Cai and Xu, 2011).

Taking into consideration all of the above reviewed biomedical strategies, ultraflexible nanosized devices, coated with biocompatible and mechanically dynamic materials, may represent an optimal solution. Owing to their advantageous features of stiffness/compliance in a neural context, such devices seem to be able to significantly attenuate the intraneural invasiveness, tissue strain, micromotion stress and, in turn, the chronic inflammatory response of the tissue.

It is worth noting, though, that there still remain several technical snags that must be overcome in the manufacturing of suitable nanofluidic components over the next decade. Because of its novelty, expertise in nanofluidics is not as robust as the one in microfluidics and it lacks standardized procedures for the fabrication of neural nanodevices. So, manufacturing accuracy of neural nanointerfaces still depends a lot on the ability of the single producer.

However, current fabrication technologies of advanced neural electrodes that combine the employment of new CPs with complex micro- and nano-structured configurations, such as fluidic microdrives, are based on rapidly growing micro- and nano-electronics expertise. These novel methodologies permit the development and use of flexible and small-sized devices with more targeted stimulation by applying low voltages in a safe manner. Therefore, they allow researchers to obtain very good neural signals while reducing the implant invasiveness and its mechanical deformation (Gerwig et al., 2012; Lorach et al., 2015; Samba et al., 2015; Bazard et al., 2017; Qi et al., 2017; Vitale et al., 2018). Furthermore, to match the mechanical texture of the neural tissue, especially of the brain, conductive and ultraflexible nanomaterials, such as CNTs, ultrathin films of graphene and nanowires have been explored. Such highly flexible and compliant electrodes can thus bend and adapt to the movements of the host tissue only in a slightly invasive and detrimental manner. Nevertheless, various methods for the implantation into the neural tissue of these nanofabricated devices require temporary stiffening factors that sometimes tend to augment the electrode size and stiffness, thereby increasing also tissue damage, cell death and eventually giving rise to a severe and unwanted inflammatory response (Vitale et al., 2018).

Hitherto, the majority of these studies that investigate alternative strategies against the FBR have been carried out either on the CNS or using other cell types *in* vitro, such as cardiac cells. Hence, many efforts have yet to be done to achieve suitable solutions also in peripheral nerves. Despite these hurdles, we believe that the challenge of ensuring a high-resolution release of bioactive chemicals against the FBR by minimally invasive neural interfaces while, at the same time, precisely controlling neurostimulation will need nanofluidics to be fully accomplished. Indeed, the smaller the size of the invasive electrode with an associated lower stiffness, the better the response of the neural tissue and the more selective and tailored will be the control over the device functionality. Among the main advantages for the use of nanoelectrodes, there is undoubtedly the enhanced mass transport, favored by the reduced dimensions, which determines an increased flow of Faradaic currents (Kotov et al., 2009). Another plus is represented by the increased spatial resolution of neural stimulation compared to microelectrodes (Fattahi et al., 2014), and the possibility to miniaturize several parallel nanoelectrodes within the same device, so to be used for simultaneous multiplexed measurements (Wang M. et al., 2018). Indeed, a more accurate control of the structural features of neural interfaces at subcellular level, with improved electrical properties, is preferable for neural recordings *in vivo*, while limiting detrimental side effects.

However, the chronic use of such invasive, although soft and small-sized electrodes, for neuroprosthetic applications will require biochemical functionalization of the surface with biocompatible coatings. These could be bioactive moieties (e.g., specific chemical signals from peptide epitopes) incorporated within conductive polymers, leveraging their tunable physicochemical properties that provide a wide versatility of intervention. A detailed investigation on how the nanotopography modifications and the chemical reactive potential of the surface can reduce plasma protein adsorption and immune cell adhesion will help control the inflammatory response. In addition, the controlled and continuous release of neurotrophic factors and the targeted delivery of therapeutic drugs to further improve the biological response to the implant and avoid the FBR will be of paramount importance. Accordingly, tailoring zwitterionic hydrogels to incorporate bioactive materials, such as ECM-derived organic components (e.g., RGD motifs that mediate the cell-fibronectin attachment) or neural cells will be pivotal (Von Der Mark et al., 2010). In this regard, it could be envisioned as particularly appealing a cell-based co-therapy, with the integration of autologous neural cells or patient-specific induced pluripotent stem cell (iPSC)-derived neural cells into the interface coating so to escape their recognition by the host immune system, hampering the consequent inflammatory cascade (Xu et al., 2013; Amin et al., 2016). Together with these solutions, loading the hydrogel with selected chemicals and drugs either linked to the surface or encapsulated into novel NPs seem the best routes to take (Aregueta-Robles et al., 2014; Lotti et al., 2017). Also, diffusion-mediated delivery systems based on micro-optical fluidic devices and microfluidic channels integrated into neural interfaces may represent another valid intervention strategy for a controlled release of therapeutics in chronic implants (Retterer et al., 2004; Takehara et al., 2014). These newly developed microfluidic devices have been characterized only *in vitro* and *in vivo*, though in mouse brain and in chronically implanted intracortical probes in rats, but they have been proven to be effective in controlling and hindering reactive responses in neural cells and brain tissue.

How limiting the leakage and the exhaustion of the drug payload in the microenvironment around the electrode site? This question still remains an open issue that the implementation of surface micromachining for the synthesis of NPs as ideal drug carriers, and microfluidic technologies could likely solve in the next years. For instance, integration and modification of the electrode surface through MEMS devices, microactuators and DSRs with permeable actuating membranes, may at least help control the pharmacological release and limit the drug leakage.

It is well known that biomaterials or tissue-engineered constructs can strongly influence the interactions between a foreign body and the host immune system. Therefore, the deposition technology of the most appropriate coating on the invasive electrode and its modification with biomimetic surfaces targeted to support tissue-specific cell functions will pave the way to the definitive solution for mitigating the FBR and for advancing the long-term use of neural-interfaced prostheses. It follows that, before *in vivo* testing, the choice of the best biomimetic coating to be used will rely on preliminary results gained from complex *in vitro* co-culture systems, as Lab-on-a-Chip devices. The latter should indeed be developed so as to recapitulate more closely all biological aspects of the intricate tissue damage, vascular injury and inflammatory cascade associated with electrode implantation. Thus, leveraging on complex microfluidic and, hopefully, nanofluidic co-culture platforms for mimicking both nervous tissue microenvironment, with patient-specific cell types, and the implant-induced FBR, one could preliminarily analyze the tissue response to a certain biomaterial coating in a physiologically relevant manner (Sharifi et al., 2019). Such opportunity raises the need for a strict collaboration between medical sciences and bioengineering. The former are necessary for having a detailed knowledge on specific mechanisms and timing of adsorption of the host proteins and cells on the implant surface; bioengineering expertise and technologies are instead essential to reproduce and simulate the entire environment, behavior and physiological responses of the nervous tissue to biomaterials. Additional work will be required to identify exactly and control the biological mechanisms of the wound healing process, and shed light on the causal connections between mechanical, chemical, immunological and inflammatory events underlying the acute and chronic peripheral nerve response. Notably, the important role played by the blood-nerve barrier must be better investigated, whose stability can be compromised by the traumatic event of the device insertion (Stubbs, 2020).

A further technical enrichment for increasing *a priori* our knowledge on the mechanisms underlying the development of the FBR for a more effective electrode engineering, comes from the use of *in silico* methods. In this respect, a very illuminating example showed a data-driven approach based on polynomial functions to simulate and investigate the development of scar tissue outgrowth around an implanted neural device over time (Sergi et al., 2020). Such computer-based approach could be combined with micro/nanofabrication and biochemical functionalization techniques for having a more representative prediction of the possible fibrous capsule consistency before collecting experimental data, thereby helping scientists in the choice of the most suitable surface coating against the development of the FBR (Di Pino et al., 2010). Overall, addressing these interesting challenges will require a close interaction between neuroengineering and biology on multiple levels for producing and, once inserted, stabilizing cutting-edge neural interfaces, and thus responding to the requests of the clinical therapy.

## Author Contributions

MG conceived the idea, wrote the manuscript, and prepared figures and tables. GV supervised and revised the manuscript. SG contributed to wrote the manuscript and prepared the tables. VD supervised the manuscript preparation. GD contributed to wrote, supervised, revised, and critically discussed the manuscript. All authors contributed to the article and approved the submitted version.

## Conflict of Interest

The authors declare that the research was conducted in the absence of any commercial or financial relationships that could be construed as a potential conflict of interest.
